# Cortex synthesis during *Bacillus subtilis* sporulation depends on the transpeptidase activity of SpoVD

**DOI:** 10.1111/1574-6968.12202

**Published:** 2013-07-08

**Authors:** Ewa Bukowska-Faniband, Lars Hederstedt

**Affiliations:** Department of Biology, Microbiology Group, Lund UniversityLund, Sweden

**Keywords:** peptidoglycan, penicillin-binding protein, endospore

## Abstract

The nonessential process of peptidoglycan synthesis during *Bacillus subtilis* sporulation is one model to study bacterial cell wall biogenesis. SpoVD is a class B high-molecular-weight penicillin-binding protein that is specific for sporulation. Strains lacking this protein produce spores without the peptidoglycan cortex layer and are heat sensitive. The detailed functions of the four different protein domains of SpoVD are unknown, and the observed phenotype of strains lacking the entire protein could be an indirect defect. We therefore inactivated the transpeptidase domain by substitution of the active-site serine residue. Our results demonstrate that endospore cortex synthesis depends on the transpeptidase activity of SpoVD specifically.

## Introduction

The cell wall in bacteria determines shape and provides structural support and protection to the cell. Peptidoglycan is the major structural component of the bacterial cell wall. It consists of glycan strands that are cross-linked via short peptide chains. Biosynthesis of peptidoglycan can be divided into three stages: (1) synthesis of precursor molecules in the cytoplasm, (2) transport of lipid II across the membrane and (3) incorporation of disaccharide-peptide units into nascent peptidoglycan at the outer side of the cytoplasmic membrane (Foster & Popham, 2002; Typas *et al*., 2012). The incorporation of disaccharide-peptide units is achieved through the action of penicillin-binding proteins (PBPs), which catalyze transglycosylation and transpeptidation reactions (Scheffers, 2007; Typas *et al*., 2012). Bacterial cell wall synthesis is an effective target for many antibiotics in clinical use, such as penicillins and cephalosporins (Bugg *et al*., 2011). However, peptidoglycan synthesis and cell wall morphogenesis are still far from understood at the molecular level. The complex macromolecular structure of peptidoglycan and the essential nature of many proteins involved in its synthesis make experimental studies difficult.

Peptidoglycan synthesis occurs during sporulation in *Bacillus subtilis* in the form of spore cortex synthesis that takes place in the intermembrane space of the forespore (Eichenberger, 2012). Sporulation takes several hours to complete and involves a series of morphological changes. Upon initiation of sporulation, the cell divides asymmetrically to form the smaller forespore and the larger mother cell. Subsequently, the forespore becomes engulfed by the mother cell in a phagocytosis-like process, which results in the formation of a double-membrane-enclosed forespore in the mother cell cytoplasm. Upon completion of engulfment, the cortex layer is assembled in the forespore intermembrane compartment and the multilayered protein coat is formed on the surface of the forespore. Finally, the mature spore is released via lysis of the mother cell (Piggot & Hilbert, 2004; Eichenberger, 2012).

Cortex synthesis, unlike vegetative cell wall synthesis, is not essential for cell viability and growth. Therefore, peptidoglycan synthesis during sporulation enables analysis of mutants defective in enzymes that otherwise are essential for growth and offers an experimental system to elucidate cell wall assembly. Heat resistance of spores depends on the presence of the cortex layer (Todd *et al*., 1986), and loss of heat resistance provides a convenient assay in screening for mutants defective in cortex synthesis.

SpoVD is a class B high-molecular-weight PBP that is essential for spore cortex synthesis (Daniel *et al*., 1994). SpoVD has four domains: an N-terminal single transmembrane segment (*c*. 46 residues) followed by a domain of an unknown function (*c*. 180 residues), a transpeptidase domain (*c*. 339 residues) and C-terminal PASTA domain (*c*. 60 residues). The detailed functions of the domains are unknown, but presumably the transpeptidase domain catalyzes the formation of peptide cross-links between glycan strands in nascent cortex. The activity of the transpeptidase domain of SpoVD seems regulated by a dithiol-based redox switch not previously reported for any PBP (Liu *et al*., 2010).

Previous studies on the role of SpoVD in cortex synthesis were carried out using various mutants in which the *spoVD* gene was insertionally inactivated (Daniel *et al*., 1994; Fay *et al*., 2010; Liu *et al*., 2010), leading to absence of the SpoVD protein. SpoVD is believed to form a peptidoglycan synthesis multiprotein complex together with SpoVE (Fay *et al*., 2010) and other not yet identified proteins. SpoVE is a putative lipid II flippase that recently was shown to depend on SpoVD for stability against degradation in sporulating cells (Fay *et al*., 2010). SpoVE- and SpoVD-deficient *B. subtilis* mutants show identical phenotypes, that is, form heat-sensitive spores completely lacking the cortex layer. The object of the work reported here was to elucidate whether the synthesis of cortex depends on the transpeptidase activity of SpoVD or on some other function of this membrane protein.

## Materials and methods

### Bacterial strains and growth media

Used bacterial strains are listed in Table 1. *Escherichia coli* TOP10 was used to propagate plasmid DNA. *Escherichia coli* strains were grown at 37 °C in LB medium or on LB agar plates (Sambrook & Russell, 2001). *Bacillus subtilis* strains were grown at 30 or 37 °C in LB medium, nutrient sporulation medium with phosphate (NSMP) (Fortnagel & Freese, 1968), growth medium and resuspension medium for induction of sporulation (Nicholson & Setlow, 1990), Spizizen's minimal medium (SMM) (Harwood & Archibald, 1990) or on tryptose blood agar base (TBAB) plates (Difco). Antibiotics were used when appropriate at the following concentrations: ampicillin 100 μg mL^−1^ for *E. coli*, and spectinomycin 100 μg mL^−1^, erythromycin 1 μg mL^−1^, chloramphenicol 3–5 μg mL^−1^, tetracycline 15 μg mL^−1^ for *B. subtilis*. TBAB medium supplemented with 1% (w/v) soluble starch was used to test amylase activity of *B. subtilis* colonies.

**Table 1:** Strains and plasmids used in this work

Strain or plasmid	Genotype/Description	Origin/Reference
*E. coli*		
TOP10	*F- mcrA Δ(mrr-hsdRMS-mcrBC) φ80lacZΔM15 ΔlacX74 nupG recA1 araD139 Δ(ara-leu)7697 galE15 galK16 rpsL(Str*^*R*^*) endA1 λ*^*−*^; Str^R^	Invitrogen
*B. subtilis*		
1A1	*trpC2*	BGSC*
LMD100	*trpC2 spoVDΩ*pLEB2; Cm^R^	This work
LMD101	*trpC2* Δ*spoVD*	This work
LMD104	*trpC2* Δ*spoVD amyE::P*_*spoVD*_*-spoVD-mCherry*; Sp^R^	This work
LMD115	*trpC2* Δ*spoVD amyE:: P*_*spoVD*_ *-spoVD(Ser294Ala)-mCherry;* Sp^R^	This work
Plasmids		
pJM103-I-SceI	Suicide integration vector pJM103 (Perego, 1993) with I-SceI restriction site; Ap^R^, Cm^R^	Perego (1993)
pBKJ223	I-SceI expression vector; Ap^R^, Tc^R^	Janes & Stibitz (2006)
pDG1730	*amyE* integration vector; Ap^R^, Sp^R^, Ery^R^	Guerout-Fleury *et al*. (1996)
pKS-mCherry-E-T3	*E. coli* vector for generating gene fusions with mCherry; Ap^R^	N. Ausmees
pLEB1	289 bp region upstream of *spoVD* cloned into pJM103-I-SceI; Ap^R^, Cm^R^	This work
pLEB2	336 bp region downstream of *spoVD* cloned into pLEB1; Ap^R^, Cm^R^	This work
pLEB5	pKS-mCherry-E-T3 with a 2.0-kb fragment containing *P*_*spoVD*_ *–spoVD*	This work
pLEB6	pDG1730 with a 2.8-kb fragment containing *P*_*spoVD*_ *-spoVD-mCherry* gene fusion; Ap^R^, Sp^R^, Em^R^	This work
pLEB19	pDG1730 with a 2.8-kb fragment containing *P*_*spoVD*_ *-spoVD(Ser294Ala)-mCherry*; Amp^R^, Sp^R^, Em^R^	This work

* Bacillus Genetic Stock Center, Columbus, OH.

Ap, ampicillin; Cm, chloramphenicol; Em, erythromycin; Sp, spectomycin, Str, streptomycin; Tc, tetracycline.

### DNA techniques

DNA manipulation was performed by standard methods (Sambrook & Russell, 2001). Plasmid DNA from *E. coli* was isolated using the Quantum Miniprep (BioRad) or QIAfilter Midi (QIAGEN) plasmid purification kit. Chromosomal DNA from *B. subtilis* was isolated according to the procedure described by Marmur (Marmur, 1963). PCR was carried out using Phusion high-fidelity DNA polymerase (Finnzymes). Supporting Information, Table S1, shows the sequences of oligonucleotides used to amplify DNA using either *B. subtilis* chromosomal DNA or plasmid DNA as template. DNA ligation was performed using T4 DNA ligase (New England Biolabs) at 14 °C, over night. Ligates were precipitated prior to transformation into *E. coli* by electroporation (Hanahan *et al*., 1991). *Bacillus subtilis* was grown to natural competence, as described by Hoch (Hoch, 1991), and *c*. 0.5 μg of DNA was added to 0.5 mL competent cells. All DNA fragments cloned in plasmids were verified by sequence analysis.

### Construction of plasmids

Used plasmids are listed in Table 1.

#### Construction of pLEB2

This plasmid was constructed in two steps. First, a fragment containing the *c*. 280 bp upstream region of *spoVD* and the three-first nucleotides of the *spoVD* open reading frame was amplified by PCR using primers Ewa1 and Ewa2 and *B. subtilis* 1A1 chromosomal DNA as template. Primers Ewa1 and Ewa2 generated restriction sites for XmaI and BamHI, respectively. Following restriction enzyme digestion, the PCR product was ligated into pJM103-I-SceI cut with the same enzymes, resulting in plasmid pLEB1. Next, a fragment containing the three last nucleotides of *spoVD* and the *c*. 330 bp downstream region of *spoVD* was amplified using primers Ewa3 and Ewa4. The PCR product was digested with BamHI and SphI and inserted into pLEB1 cut with the same enzymes, resulting in plasmid pLEB2.

#### Construction of pLEB6

Primers Ewa9 and Ewa10 were used to amplify a 2068-bp fragment of the *B. subtilis* 1A1 chromosome comprising the promoter region and the coding sequence of *spoVD* (without the stop codon). These primers introduced restriction sites for KpnI and XhoI. The PCR fragment was digested and inserted into KpnI/XhoI-digested pKS-mCherry-E-T3, resulting in plasmid pLEB5. The resulting plasmid encodes a SpoVD-mCherry fusion protein with a linker (LEVDGIDKLDDP). The *P*_*spoVD*_*-spoVD-mCherry* in-frame gene fusion was amplified from pLEB5 with primers Ewa5 and Ewa13, generating a 2800-bp fragment flanked by EcoRI and BamHI sites. After digestion with EcoRI and BamHI, the PCR product was cloned into EcoRI/BamHI-digested pDG1730, giving plasmid pLEB6.

#### Construction of pLEB19

The codon for the active-site serine (Ser294) residue of the transpeptidase domain of SpoVD in pLEB6 was changed to encode alanine by site-directed mutagenesis (Phusion® Site-Directed Mutagenesis, Finnzymes) using primers Ewa30 and Ewa31, resulting in plasmid pLEB19.

### Construction of *B. subtilis* strains

All *B. subtilis* strains described in this work are derivatives of 1A1 (Table 1). *Bacillus subtilis* LMD101 deleted for *spoVD* was constructed based on a method described for *Bacillus anthracis* by Janes and Stibitz (Janes & Stibitz, 2006) with slight modifications. Briefly, pLEB2 (carrying a I-SceI restriction site) was transformed into *B. subtilis* 1A1, resulting in the integration of the entire plasmid at the *spoVD* locus by a single crossover event (Campbell-type recombination). The obtained *B. subtilis* strain, LMD100, was then transformed with pBKJ223 which encodes the I-SceI endonuclease. SceI cleavage generates a double-stranded break in the chromosomal DNA, which can be repaired by homologous recombination resulting in our case in either a markerless deletion of *spoVD* or retained *spoVD*. Tetracycline-resistant transformants were scored for loss of the allelic exchange plasmid by patching single colonies onto TBAB plates containing chloramphenicol. Chloramphenicol-sensitive clones were then passed two times on TBAB plates without tetracycline in order to lose pBJ223. Chromosomal DNA was isolated from transformants that were chloramphenicol- and tetracycline-sensitive. The *spoVD* in-frame deletion was confirmed by PCR (using primers Ewa1 and Ewa4) and DNA sequence analysis. The constructed strain was named LMD101. *Bacillus subtilis* LMD101 was transformed with pLEB6 and pLEB19 resulting in strains LMD104 and LMD115, respectively.

### Light microscopy

A 100 μl sample was taken from the culture of sporulating cells. The cells were collected by centrifugation and suspended in such a volume of phosphate-buffered saline that the density of cells was appropriate for microscopy. Five microlitre of the cell suspension was added on an agarose pad on a microscopy glass slide and covered with a cover slip. Phase contrast and fluorescence images were acquired using a Zeiss Axio Imager.Z1 microscope equipped with X-Cite 120 Illumination (EXFO Photonic Solutions Inc.) and a 9100-02 EM-CCD camera (Hamamatsu Photonics). Eight hundred millisecond exposure time was typically used to obtain fluorescence images of cells. Finally, images were processed in Volocity 5.5 and saved in TIFF format.

### Immunoblot analysis

Proteins were fractionated by SDS-PAGE and transferred to a PVDF blotting membrane (Immobilon P, Millipore) using a wet blot. Transfer buffer was 20 mM Tris, 150 mM glycine buffer containing 20% (v/v) methanol. Anti-dsRed antiserum from rabbit (Clontech) was used at a dilution of 1 : 1000. Immunodetection was carried out by chemiluminescence using anti-rabbit secondary antibodies conjugated to horseradish peroxidase (GE Healthcare) in 1 : 3000 dilution and Super Signal® West Pico Chemiluminescent substrate (Pierce Chem. Co.).

### Spore assay

Spores were prepared either by growth in NSMP medium as described by Erlendsson *et al*. (2004) or by the resuspension method (Nicholson & Setlow, 1990). Sporulation efficiency was analyzed by heating 5 mL of the culture at 80 °C for 10 min. Serial dilutions of heated and unheated samples were spread on TBAB plates. The number of colonies was counted after incubation of the plates at 37 °C over night, and the spore yield was calculated.

### Bocillin FL-binding assay

The Bocillin FL-binding assay to detect PBPs was performed as described before (Zhao *et al*., 1999) with the following modifications. Membrane samples containing 3 mg mL^−1^ of proteins were incubated with 25 μM Bocillin FL (Invitrogen) for 30 min at 35 °C. The reaction was stopped by adding equal volume of 2× SDS sample buffer followed by incubation of samples for 30 min at 40 °C. The proteins (30 μg in each well) were separated by SDS-PAGE on a 8% (w/v) polyacrylamide gel. Bocillin FL-labeled PBPs were detected using a ChemiDoc™ MP Imaging System (Bio-Rad) (excitation light 455–485 nm, emission light filter 532AE28). Subsequently, the gel was stained with Coomassie Brilliant Blue in order to confirm equal amount of proteins loaded in each well.

### Miscellaneous methods

Electron microscopy of endospores was performed as described before (Erlendsson *et al*., 2004).

Membranes from cells collected at different stages of sporulation were isolated as described previously (Hederstedt, 1986). Protein concentrations were determined using the BCA Protein Assay Kit (Thermo Scientific).

## Results and discussion

### Construction and complementation of an in-frame *spoVD* deletion in the chromosome of *B. subtilis*

**Figure 1:**
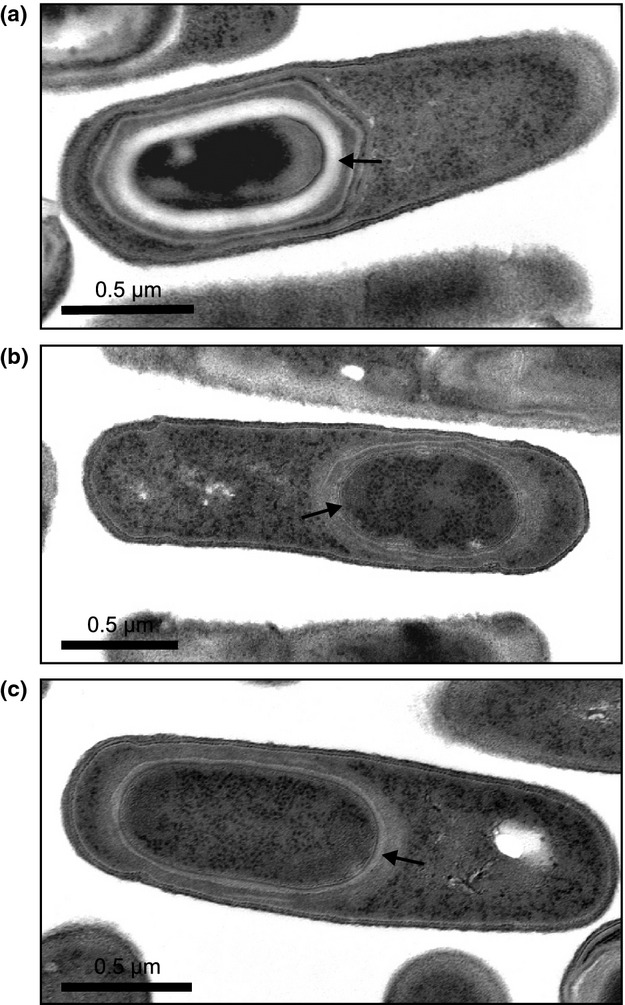
Electron micrographs showing the morphology of the *Bacillus subtilis* endospore in the mother cell of strains: (a) LMD104 (wild-type SpoVD-mCherry), (b) LMD101 (lacks SpoVD) and (c) LMD115 (contains Ser294Ala substitution in SpoVD-mCherry). The cortex layer in the spore of LMD104 is indicated by an arrow. In the case of the mutant strains, an arrow indicates lack of cortex. Scale bar is 0.5 μm.

For convenient analysis of *spoVD* mutant alleles in *B. subtilis,* we first constructed a strain, LMD101, which lacks the entire ORF of the *spoVD* gene. The *spoVD* gene lies in a cluster of genes involved in cell wall synthesis (Daniel & Errington, 1993; Daniel *et al*., 1994). Strain LMD101 contains a markerless in-frame *spoVD* deletion to avoid possible polar effects on downstream genes. Strain LMD101, as expected, forms heat-sensitive spores (Table 2) without cortex layer (Fig. 1b).

**Table 2:** Sporulation efficiency of *B. subtilis* strains

Strain	Relevant property	CFU mL^−1^ preheat	CFU mL^−1^ postheat	% sporulation*
1A1	Wild type	3.5 × 10^8^	2.7 × 10^8^	76
LMD101	SpoVD^−^	1.7 × 10^7^	< 10	< 6 × 10^−7^
LMD104	SpoVD^−^, SpoVD-mCherry	1.1 × 10^9^	6.7 × 10^8^	55
LMD115	SpoVD^−^, SpoVD(Ser296Ala)-mCherry	1.7 × 10^8^	< 10	< 6 × 10^−8^

* Cells were grown in NSMP medium for 2 days at 37 °C. Heat resistance of cells was assayed by incubation at 80 °C for 10 min. Sporulation efficiency was calculated as colony-forming units (CFU) after heating the culture divided by CFU of not heated culture. Sporulation assays were performed at least three times per strain. Representative results are shown.

**Figure 2:**
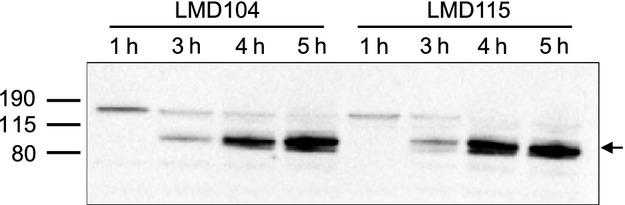
Immunoblot for SpoVD in *Bacillus subtilis* strains LMD104 (wild-type SpoVD-mCherry) and LMD115 (active-site SpoVD-mCherry mutant). Cells were sporulated by resuspension at 30 °C, and samples were taken at hourly intervals after the resuspension as indicated. Isolated membranes, 15 μg of proteins, were loaded in each lane. The samples were probed with anti-dsRed serum. SpoVD-mCherry (*c*. 100 kDa) is indicated by an arrow. The antigen band seen at > 115 kDa is background independent of mCherry. Molecular mass markers, in kDa, are indicated.

To complement the deletion mutant and to study the subcellular localization of SpoVD, a C-terminal translational fusion of mCherry to SpoVD was constructed. The gene fusion was placed under the native *spoVD* promoter and inserted into the *amyE* locus of strain LMD101. The obtained strain, LMD104, formed heat-resistant spores (Table 2) with normal cortex layer (Fig. 1a) showing that the fusion protein is functional. Presence of the full-length fusion protein (*c*. 100 kDa) in membranes was confirmed by immunoblot analysis with antibodies directed against mCherry (Fig. 2, strain LMD104). No degradation of fusion protein, for example, cleavage between SpoVD and mCherry, was detected in cell extracts using immunoblot (data not shown).

### Presence of endospore cortex is dependent on the transpeptidase activity of SpoVD

To determine whether the lack of cortex in *spoVD*-negative mutants is an indirect effect, for example, a result of disruption of a protein complex (where SpoVD might act as a scaffold for other proteins), a result of SpoVE degradation or is due to lack of SpoVD enzyme activity, we mutated the active-site serine residue of the transpeptidase domain in SpoVD. This serine is essential for transpeptidase activity (Goffin & Ghuysen, 1998).

Plasmid pLEB6, which carries the *spoVD-mCherry* gene fusion, was used as a template for site-directed mutagenesis, and the codon for the active-site serine (Ser294) was changed to encode alanine. The mutant gene variant was inserted into the *amyE* locus in the chromosome of strain LMD101. The obtained SpoVD-mCherry active-site mutant strain, LMD115, was found to form heat-sensitive spores (Table 2). Electron microscopic examination of LMD115 sporulating cells showed lack of the cortex layer in spores (Fig. 1c), consistent with the heat sensitivity of the spores.

**Figure 3:**
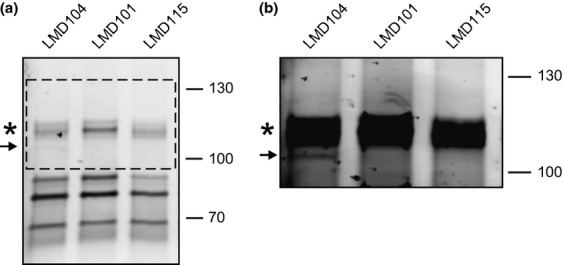
Covalent binding of the fluorescent penicillin derivative Bocillin FL to PBPs present in sporulating *Bacillus subtilis* cells. (a) Membranes from strains LMD104 (wild-type SpoVD-mCherry; positive control), LMD101 (SpoVD deletion mutant; negative control) and LMD115 (active-site SpoVD-mCherry mutant). Cells were sporulated by resuspension at 30 °C, and membranes were isolated from cells at 5 h of sporulation (see Fig. 2). After incubation with Bocillin FL, membrane proteins were separated by 8% SDS-PAGE. The electrophoresis was run to resolve high-molecular-weight proteins. The gel was analyzed by fluorometry. (b) shows an overexposure of the area outlined by the dashed box in panel (a) in order to reveal the relatively weak fluorescence intensity of the band corresponding to SpoVD-mCherry in LMD104. The position of Bocillin FL-labeled SpoVD-mCherry is indicated by an arrow. An asterisk indicates the position of PBP1a/b identified on the basis of published data (McPherson *et al*., 2001). Molecular mass markers, in kDa, are indicated on the right-hand side of the panels.

Normal temporal expression and presence of the full-length mutant fusion protein (*c*. 100 kDa) in the membrane fraction of sporulating LMD115 cells was confirmed by immunoblot analysis (Fig. 2, strain LMD115). Next, the enzymatic activity of the mutant protein was tested using Bocillin FL, a commercially available fluorescent penicillin derivative (Zhao *et al*., 1999). Isolated membranes of LMD104 and LMD115 were incubated with Bocillin FL, and proteins were fractionated by SDS-PAGE. Strain LMD104 showed a weakly fluorescent protein band corresponding to SpoVD-mCherry (Fig. 3). As expected, the mutant SpoVD-mCherry protein of LMD115 was unable to covalently bind Bocillin FL, confirming that transpeptidase activity was abolished by the Ser294Ala mutation.

### Subcellular localization of mutant SpoVD

**Figure 4:**
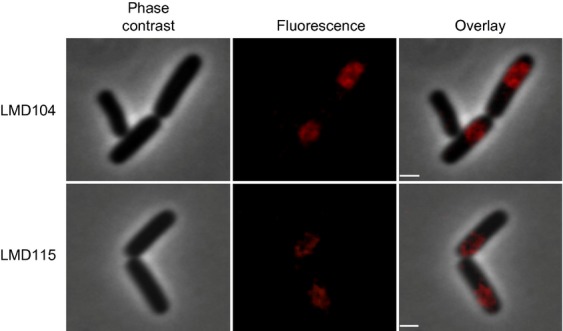
Subcellular localization of wild-type (LMD104) and Ser294Ala mutant (LMD115) SpoVD-mCherry in *Bacillus subtilis* sporulating cells. Cells were sporulated by resuspension at 30 °C, and phase contrast and mCherry fluorescence images of cells were taken at 5 h after the start of sporulation. Scale bar is 1 μm.

Subcellular localization of some PBPs is reported to be dependent on their transpeptidase activity (Pinho & Errington, 2005; Costa *et al*., 2008). We therefore asked whether the lack of cortex in strain LMD115 is a result of mislocalization of the mutant SpoVD protein. The subcellular localization of the SpoVD(Ser294Ala)-mCherry in sporulating *B. subtilis* cells was compared with that of the wild-type variant using fluorescence microscopy. The fluorescence signal of both SpoVD(Ser294Ala)-mCherry and the functional SpoVD-mCherry was enriched at the forespore (Fig. 4). The results demonstrate that the localization of SpoVD to the forespore is not dependent on its transpeptidase activity, and the lack of cortex in the SpoVD(Ser294Ala) mutant is not a result of mislocalization of SpoVD.

## Conclusion

Our major finding is that the presence of cortex in *B. subtilis* endospores is dependent on SpoVD transpeptidase activity specifically. This suggests that other enzymes with transpeptidase activity that are expressed during sporulation, that is, Pbp2d and Pbp4b, (Pedersen *et al*., 2000; McPherson *et al*., 2001; Wei *et al*., 2004), cannot compensate for lack of SpoVD transpeptidase activity. One should note that the endospore germ cell wall layer, situated underneath the cortex layer in spores (not seen in our electron micrographs), is formed also in SpoVD-defective mutants because the heat-sensitive spores germinate normally. This layer seemingly forms during engulfment (Tocheva *et al*., 2013). The detailed role of SpoVD transpeptidase activity in cortex synthesis remains unknown. SpoVD could, for example, bind lipid II delivered across the outer forespore membrane by the activity of SpoVE and then in cooperation with other PBPs with transglycosylase activity incorporate the delivered disaccharide-peptide unit into a nascent peptidoglycan strand as previously pointed out by Fay and coworkers (Fay *et al*., 2010).

## Supporting Information

Table S1 Oligonucleotides used in this work.
