# Supplementary material for: Cortex synthesis during Bacillus subtilis sporulation depends on the transpeptidase activity of SpoVD
Source: FEMS Microbiol Lett. 2013 Jul 8;346(1):65–72. doi: 10.1111/1574-6968.12202 (PMC3906833; doi:10.1111/1574-6968.12202)
Supplement: Table S1 — Oligonucleotides used in this work. [file fml0346-0065-sd1.doc]

**Supporting Information**

# Cortex synthesis during *Bacillus subtilis* sporulation depends on the transpeptidase activity of SpoVD

Ewa Bukowska-Faniband^1^ and Lars Hederstedt

Microbiology Group, Department of Biology, Lund University, Sweden

**Table S1.** Oligonucleotides used in this work

| **Name** | **Sequence^a^** | **Restriction site/mutation** |
| --- | --- | --- |
| Ewa1 | 5’GGCCAT**CCCGGGA**GAAGAGAGGTTCT 3’ | XmaI |
| Ewa2 | 5’GGCCAT**GGATCC**AAGAGACCGTTCAC 3’ | BamHI |
| Ewa3 | 5’GGCCAT**GGATCC**TGATTCGGGCTGCCT 3’ | BamHI |
| Ewa4 | 5’GGCCAT**GCATGC**CCTTTTACACATAC 3’ | SphI |
| Ewa5 | 5’ GGCCAT**GAATTC**GTTATGCAGGCTT 3’ | EcoRI |
| Ewa9 | 5’ GGCCAT**GGTACC**GTCGTTATGCAGG 3’ | KpnI |
| Ewa10 | 5’ GGCCAT**CTCGAG**ATCGGCTGCCTCCT 3’ | XhoI |
| Ewa13 | 5’ GGCCAT**GGATCC**TTACTTGTACAGCTCG 3’ | BamHI |
| Ewa30 | 5’ PHO-GTATGAACCGGGAGCCACCTTTAAAATT 3’ | Ser294→Ala |
| Ewa31 | 5’ PHO-GTGCTCCATACTGGCAGATTTCG 3’ | - |

^a^ Introduced restriction sites are in boldface, and mutated nucleotides are underlined.
